# Arithmetic on Your Phone: A Large Scale Investigation of Simple Additions and Multiplications

**DOI:** 10.1371/journal.pone.0168431

**Published:** 2016-12-29

**Authors:** Federico Zimmerman, Diego Shalom, Pablo A. Gonzalez, Juan Manuel Garrido, Facundo Alvarez Heduan, Stanislas Dehaene, Mariano Sigman, Andres Rieznik

**Affiliations:** 1 Laboratorio de Neurociencia, Universidad Torcuato Di Tella, Buenos Aires, Argentina; 2 Departamento de Ingeniería Biomédica, Facultad de Ingeniería, Universidad de Buenos Aires, Buenos Aires, Argentina; 3 Departamento de Física, Facultad de Ciencias Exactas y Naturales, Universidad de Buenos Aires, Buenos Aires, Argentina; 4 Consejo Nacional de Investigaciones Científicas y Técnicas (CONICET), Buenos Aires, Argentina; 5 El Gato y La Caja, Buenos Aires, Argentina; 6 Cognitive Neuroimaging Unit, INSERM, Université Paris-Sud, Université Paris-Saclay, NeuroSpin center, Gif/Yvette, France; 7 Collège de France, Paris, France; Universiteit Gent, BELGIUM

## Abstract

We present the results of a gamified mobile device arithmetic application which allowed us to collect vast amount of data in simple arithmetic operations. Our results confirm and replicate, on a large sample, six of the main principles derived in a long tradition of investigation: size effect, tie effect, size-tie interaction effect, five-effect, RTs and error rates correlation effect, and most common error effect. Our dataset allowed us to perform a robust analysis of order effects for each individual problem, for which there is controversy both in experimental findings and in the predictions of theoretical models. For addition problems, the order effect was dominated by a max-then-min structure (i.e 7+4 is easier than 4+7). This result is predicted by models in which additions are performed as a translation starting from the first addend, with a distance given by the second addend. In multiplication, we observed a dominance of two effects: (1) a max-then-min pattern that can be accounted by the fact that it is easier to perform fewer additions of the largest number (i.e. 8x3 is easier to compute as 8+8+8 than as 3+3+…+3) and (2) a phonological effect by which problems for which there is a rhyme (i.e. "seis por cuatro es veinticuatro") are performed faster. Above and beyond these results, our study bares an important practical conclusion, as proof of concept, that participants can be motivated to perform substantial arithmetic training simply by presenting it in a gamified format.

## 1. Introduction

Smartphones, with more than 3 billion users across the world [1], present a unique opportunity to collect massive data in cognitive science experiments. The validity of these large scale datasets has been partly verified by demonstrating that they can afford millisecond timing precision on both stimuli and responses [2] and replications of some classic psychological paradigms [2, 3].

Here we investigate the capacity of this experimental approach to inquire about the cognitive mechanisms of arithmetic. Our aims are twofold, first to show that the main observations derived from a long tradition of laboratory based experimentation in arithmetic can be replicated with a rapid massive strategy of data acquisition. Second, to inquire whether big data in arithmetic may help us understand order effects in arithmetic that could not be reliably observed before in laboratory settings.

To these aims, we developed Moravec, an Android OS app that allows users to perform different types of operations, from 1-digit additions to 4-digits squares. The name Moravec is in honor of Hans Moravec´s paradox that states that high-level reasoning, including arithmetic with large numbers, requires very little computation, but low-level sensory and instead motor tasks often require vast computational resources. One key aspect of this investigation was to gamify the app (structure of levels of difficulties, the flow and graphic interface) to engage participants—who play at their own pace and without receiving any monetary compensation.

Two weeks after the app release, and without any major effort of diffusion, a total of 513 subjects had solved 90.000+ problems. These numbers are one order of magnitude higher than those reached in previous experiments in the area, and are constantly growing because of the possibility to participate anytime anywhere without the need of an investigator to be present.

## 2. Design and Diffusion of the Game

### 2.1. Design

The app presents different arithmetic operations which include:

Additions: two 1-digit numbers (e.g. 5 + 9) and two 2-digit numbers (e.g. 34+ 49).
Two numbers multiplications: 1-digit by 1-,2-,3-, and 4- digit numbers (e.g. 9x4, 86x8, 806x2, 3214x8).
Squares: numbers between 11 and 9999 (e.g. 21^2^, 872^2^, 7002^2^).

**Fig 1:**
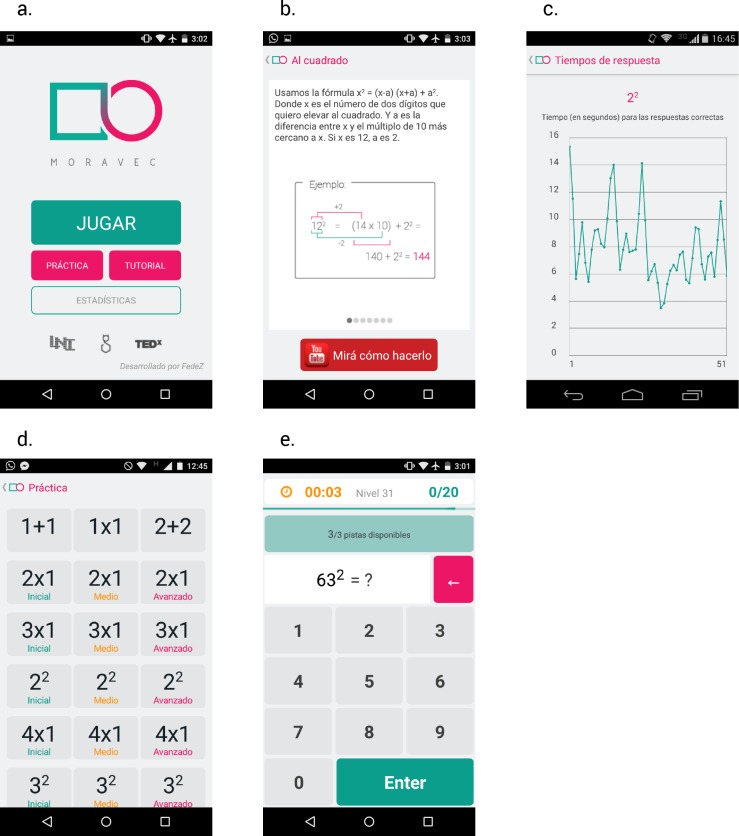
Some Moravec screenshots. (a) Initial screen with the logo and four buttons: Play (“Jugar”), Practice (“Práctica”), Tutorial and Statistics (“Estadisticas”). (b) One of the many tutorial screens explaining the algorithms and with a link to a youtube tutorial video. (c) One of the statistics shown. It plots RTs as a function of the trial number for 2-digits square operations. (d) In the “Practice” section the user can choose which operation to train. For each operation three difficulty levels can be chosen: initial, medium and advanced. In the advance practice, problems are shown for 5 seconds and then disappear while in the other cases its remains visible until the user press enter (see (e)).

These are all the atomic operations required to square a 4-digit number, using the algorithm described by the expert calculator Arthur Benjamin [4]. So, the end state of the game, and the long term goal of the activity is to train participants to perform 4-digit square calculations. This serves as a motivation since it is quite impressive to a naïve observer, and can be reached with extensive practice. The home screen (in Spanish) offers four possibilities (Fig 1): Play (“Jugar”), Practice (“Práctica”), Tutorial and Statistics (“Estadísticas”).

In the Play section, users go through 150 levels, starting with simple calculations and moving on to higher levels with more difficult problems, the most difficult task being to square 4 digits numbers. In each level 20 problems are presented and in order to advance to the next level 15 correct answers are required. If the answer is correct but it takes too much time (the exact duration depends on the level), this problem is neglected.

The Tutorial section (Fig 1B) explains the algorithm recommended for performing multiplications (left to right) and to square numbers (using the formula x^2^ = (x-a)*(x+a)+a^2^). There is a link to tutorial videos (a YouTube’s playlist called “Tutorial Moravec”) created specifically for the app, explained by author AR who is an expert mental calculator.

In the Statistics section (Fig 1C), users can visualize their progress in each type of operation. Their average RTs, error rates, and a graph showing RTs as a function of the trial number for correct answers are displayed for each type of operation. This was included as part of the app to engage participants by being able to monitor their progress.

In the “Practice” section the user can choose one operation to train, regardless of the default flow of the game (Fig 1D).

### 2.2. Game flow

**Fig 2:**
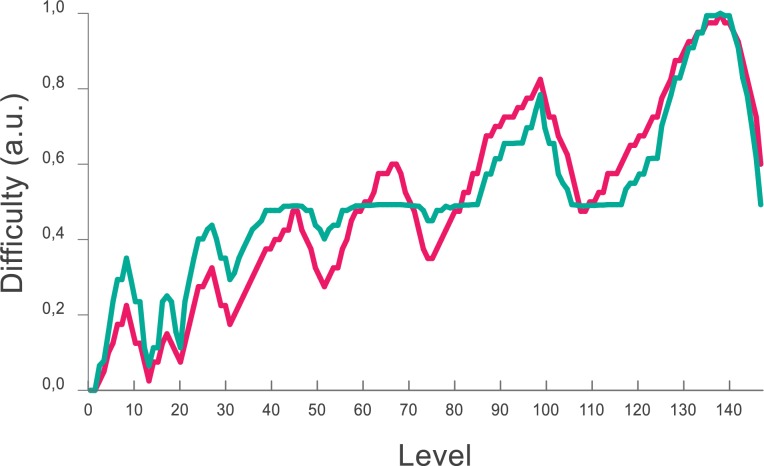
Difficulty as a function of the level. To generate an engaging experience, difficulty progresses as a series of increases and decreases. The difficulty in each level is shown according to a prediction based on the number of mental computations and memory load required by each problem (magenta). This correlates as expected with the measured RTs (green) after the experiment.

Levels in the “Play” mode are organized according to standard gaming recommendations [5]. In order to generate an engaging gameflow, difficulty evolved with a non-linear function that alternated a series of challenging ramps of increasing difficulty, followed by valleys that decrease stress and generate confidence (Fig 2).

### 2.3. Diffusion of the game

One remarkable characteristic in this study is that we reached substantial amounts of data with a very modest diffusion strategy. Moravec was released with only two posts in the personal Facebook pages of two of the authors (AR and FZ) and a third post in a private Facebook group, with 150 members, created by some of the authors as a science popularization project.

We also created a Facebook Group in which during the weeks of the experiment we updated news and pushed users to continue using the app. We identified the ones with better scores and published their result to enthusiasm users.

## 3. Ethic Statement

All subjects were informed that their data were to be collected via internet through their mobile devices and used for research. The consent was obtained before playing the game's first level: a pop-up window was launched and participants had to accept that their data would be used for research. This consent procedure was approved by the ethic committee. All the experiments described in this paper were reviewed and approved by the ethics committee: “Comité de Ética del Centro de Educación Médica e Investigaciones Clínicas “Norberto Quirno” (CEMIC)” qualified by the Department of Health and Human Services (HHS, USA): IRb00001745—IORG 0001315. The raw data and the R code developed for the mixed model analysis are available for download at https://figshare.com/articles/moravec_data_rar/4288877. The apps' codes are available at https://github.com/FedeZimmer/MoravecAndroid.

## 4. User Demographics

When opening the app for the first time, an optional form is presented to the users. Personal information such as birthdate, gender and education-level achieved were asked. From the 513 total users, 448 completed the form: 291 were male (65%), the ages ranged from 12 to 65 years old (M = 26.1, SD = 8.7), 367 subjects had completed high school (82%) and 125 had a university degree (28%). Argentineans represented 88% of the subjects, while the other 12% were from Mexico, Colombia, US and others.

**Fig 3:**
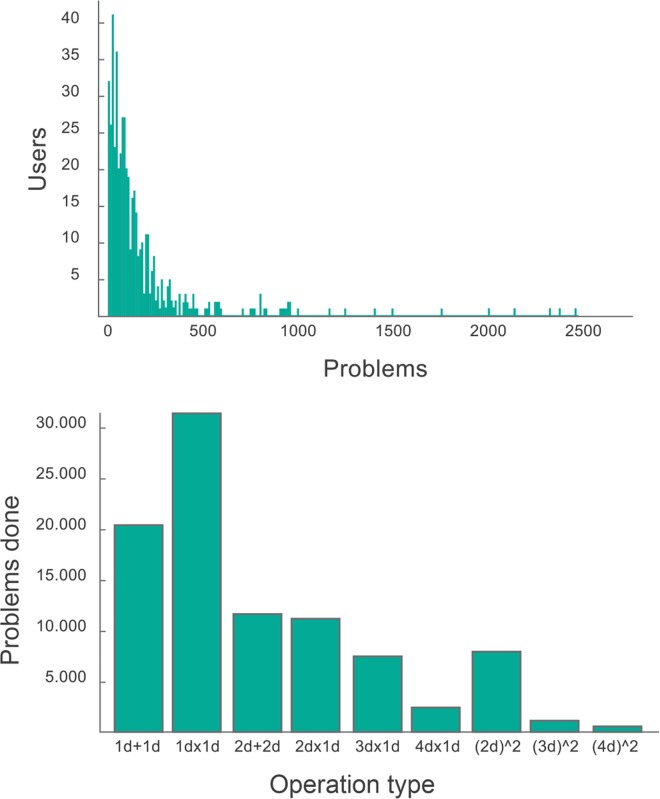
(above) number of problems solved by each user (intervals of 10 problems), and (below) total number of problems solved (by all users) by type of problem.

We show in Fig 3 histograms of the number and type of problems performed during the first two weeks after release. They show that most users performed less than 100 problems. These users played only in the first levels with easy problems. A considerable number (+300) solved at least 200 problems, and six users got really engaged and solved more than 2000 problems. These six users spent many hours per day solving arithmetic problems and were after this process able to perform 3-digit squares operations in less than one minute after two weeks of practice. This degree of calculation is typically considered as part of the repertoire of an expert mental calculator [6].

## 5. Reproducing canonical results of mental arithmetic in remote experiments

### 5.1. Review of canonical results in mental calculation and the IN model

#### 5.1.1. Response Times (RTs)

For each trial, we define the response time as the time elapsed between the visual presentation of the problem and the response of the enter key. Errors, response times that exceeded 4 standard deviations of the mean (calculated per operation type) and problems in which participants erased a digit were not included in this analysis. We considered for this analysis only operations with 1 digit by 1 digit (additions and multiplications).

The most widely and consistent reported effects which can account for the variance in RTs in one digit additions and multiplications are [7, 8]:

1. The *size effect*, the fact that reaction times (RTs) are longer for problems with larger addends or multipliers, and hence with larger responses
2. The *tie effect*, the fact that problems with identical addends or multipliers (e.g. 4x4, 3+3) are solved more quickly.
3. The *size x tie interaction effect*, which shows that the tie effect increases for larger addends or multipliers.
4. The *five effect*, the fact that one digit multiplications with an operand of 5 are solved more quickly than would be predicted on the basis of their size.

An *order effect* has also been reported [9, 10] (but see [11] where this effect has not been found): the fact that RTs depend on the order of presentation (e.g. RTs for 4 x 7 and 7 x 4).

#### 5.1.2. Error Effects

All the effects that increase RTs also result in an increase in error rates. In addition, there is a specific effect on the pattern of errors in multiplications: responses are more likely to be correct answers for problems nearby in the table. For instance, 6 x 8 = 48 is more likely to be answered as 42 (6 x 7) or 54 (6 x 9), than 49 or 47, i.e., table-distance are more common than numerical-distance errors.

#### 5.1.3. The interacting neighbors (IN) model

After substantial theoretical debate, one of the models that provides a better explanation of the data for multiplications is the connectionist model of retrieval introduced by Vergus and Fias in 2005 [8], in which learning and performance are governed by the consistency of a problem’s correct product with neighboring products in the times table. Their model introduced a critical new assumption: whether or not a product shares a common decade or unit value with neighboring products in the times table has a major impact on learning and performance. Because of this, the model is named the interacting neighbors (IN) model. This neighborhood consistency assumption appears to provide a simple and elegant explanation for the diverse phenomena of multiplication memory discussed above.

Two recent articles provide further support for the IN model [9, 12]. In particular, reference [9] shows the feature of cross-culture in the IN model: unlike those used in the West, a typical Chinese multiplication table includes only smaller-operand-first entries (e.g., 4 × 9 = 36, but not 9 × 4 = 36). Based on this unique feature, the simple multiplication for Mainland Chinese subjects has been found to show a robust operand-order effect.

It has been also shown, however, that above and beyond neighborhood-answer consistency, neighborhood-magnitude consistency also play a role [12]. This means that having neighbors similar in magnitude also affects RTs and error rates.

## 5.2. Results

We analyzed the data collected during the first two weeks after release. We used R [13]and lme4 [14] to perform a linear mixed effects analysis over it.

### 5.2.1. Size and tie effects

**Fig 4:**
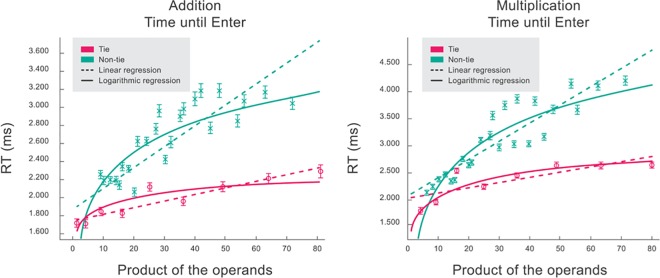
RTs for addition and multiplication in one-by-one-digit problems as a function of the product of the operands. Inter-subjects means and standard errors RTs are shown as a function of the product of the operands for tie and non-tie problems. Linear and logarithmic regression results are included.

Fig 4 shows the size, tie and size x tie effects in both addition and multiplication tables. Inter-subjects RTs means and standard errors in one digit-by-one-digit operations are shown as a function of the product of the operands (prod), which has been shown to be a good predictor for the RTs. We then performed a new model on the same data used to construct Fig 4, but including random effects: by-subject random intercepts and slopes. Results for this new model are shown Table 1. As an example, the expected RT for the operation 7+8 is, in ms, 2060 + 23.7x56 (56 is the product of the operands), i.e., 3387 ms.

**Table 1:** Results of the mixed model linear regressions for RTs using the product of the operands as independent variable.

ADDITION	intercept [ms]	std error	t-value	slope [ms/u]	Slope std error	t-value
**Different Operands**	2060	36	58	23.7	0.4	56.4
**Tie Problems**	1868	32	58	7.2	0.6	13.2
**MULTIPLICATIONS**	**intercept [ms]**	**std error**	**t-value**	**slope [ms/u]**	**Slopestd error**	**t-value**
**Different Operands**	2406	55	44	35.0	0.6	61.2
**Tie Problems**	2139	45	47	10.5	0.7	15.8

To perform this linear mixed effects analysis of the relationship between RTs and size, as fixed effects we entered product of the operands and their equality (tie or non-tie problems), with an interaction term to the model. As random effect, we had by-subject random intercepts and slopes. Visual inspection of residual plots did not reveal any obvious deviations from homoscedasticity or normality. P-values were obtained by likelihood ratio tests of the full model with the effect in question against the model without the effect in question. We obtained p< 2.2e-16 for the three effects (size, tie and size x tie).

We analyzed and compared eight other potential regressors apart from prod, the product of the operands. The regressors are: prod, the sum of the operands, its sum squared, these three regressors in log scale, the square root of its products and its sums, and the min operand. Each regressor was implemented separately and was included as the only one independent variable to predict RTs based on size, i.e., we ran a new regression for each regressor. Models were evaluated through the Akaike Information Criterion (AIC), which is a measure of the quality of each model relative to a null model that assumes that RTs are constant and do not depend on the problem. The preferred model is the one with the minimum AIC value. Values for each model are shown in Table 2. Interestingly, the logarithm of the product is the best regressor in the times table for both tie and non-tie problems. This is easily observed by visual inspection of Fig 4, where linear and logarithmic fits are shown. In the addition table, the root square of the product is better than the logarithm in the non-tie case, while a linear regressor best fits the data in the tie case.

**Table 2:** Linear mixed effects analysis for RTs for addition and multiplication and for tie and nontie problems, using eight different regressors: the product of the operands (prod), the sum of the operands (sum), its sum squared (sum^2), these three regressors in log scale (log(prod), log(sum), and log(sum^2)), the square root of its products (sqrt(prod)) and its sums (sqrt(sum)), and the min operand. Bold letters indicate best fit, i.e. minimum AIC value. Observe that in the tie cases, the min, sqrt(prod) and sum regressors represent the same condition.

		prod	Sum	sum^2	log(prod)	log(sum)	log(sum^2)	sqrt(prod)	sqrt(sum)	Min
MULTIPLICATION	nonties	314488	314518	314563	**314258**	314705	314705	314265	314581	314687
	ties	52903	52862	52903	**52828**	**52828**	**52828**	52862	528843	52862
ADDITION	nonties	279525	279558	279607	279386	279905	279905	**279261**	279676	279649
	ties	34505	**34493**	34503	34508	34508	34508	**34493**	34496	**34493**

#### 5.2.2. Five-effect and per-operand analysis

**Fig 5:**
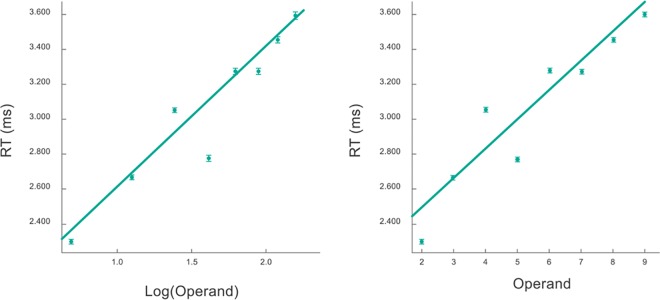
Mean RT per operand for the times table in linear (right) and logarithmic (left) scales. Linear regressions show that the data is best fitted in log scale. The mean RT for the operand 5 is neglected in the regressions. Error bars are barely seen due to the large number of collected data.

In Fig 4 it is clear that above and beyond the size and tie effects, there are other factors that account for the variance. We looked at whether some of this can be accounted by specific operands effects (Fig 5). This analysis reveals a *five-effect*, which is clearly observed in these plots. To quantify it, we performed a linear mixed effects analysis in which as fixed effects we entered product of the operands and presence of number 5 in one of them, and as random effects we had by-subject random intercepts. P-value was obtained by likelihood ratio tests of the model with size and five effects against the model without the five effect. We obtained p< 2.2e-16. We conclude that RTs for problems with 5 are faster (mean = -319 ms, SE = 22ms, t-value = -15) than would be expected by size-effect only.

**Fig 6:**
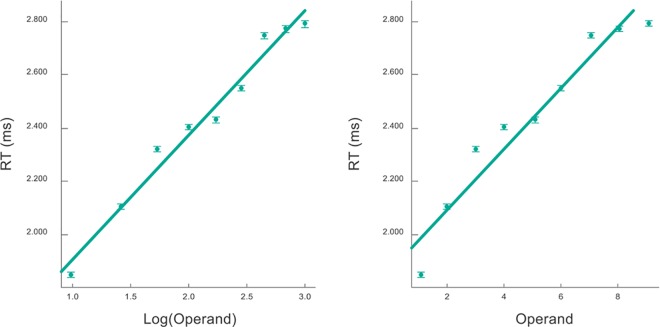
Mean RT per operand for the addition table in linear (right) and logarithmic (left) scales. Linear regressions show that the data is best fitted in log scale. Error bars are barely seen due to the large number of collected data.

We note that above and beyond the five effect, this dependence shows a saturating non-linearity. In fact, the regression of RTs as a function of the log of the different operands in the times table improved significantly using a log scale in the x axis (Fig 5): R2 = 0.98 compared to R2 = 0.91 for the linear scale, p < 0.001 according to a cox test for non-nested models. The same result was observed for additions (Fig 6): R2 = 0.98 for the log scale compared to R2 = 0.93 using a linear scale (the comparison of the cox test was also significant with p < 0.001).

#### 5.2.3. Order effects

We found significant order-effects in both addition (mean = 55ms, SE = 15 ms, t-value = 3.7) and times tables (mean = 45 ms, SE = 19ms, t-value = 2.4). By the likelihood ratio tests, including or not the fixed effect Op1>Op2 in the mixed linear model, we found in both cases that answers are faster when the maximum operand appears first (e.g. 7x4 are answered faster than 4 x 7; p = 0.02 for the times table and p = 0.0002 for the addition table). A reversed order effect for the times table had been observed before in Chinese mainland speakers, which seems to be driven by language biases, since they learn the table in only the min-max order [9, 15]. This is the first time an order effect for multiplication is observed in an occidental country (including the English-speaking world). Order effects have been observed before for addition in English speakers [10].

#### 5.2.4. Error effects

**Fig 7:**
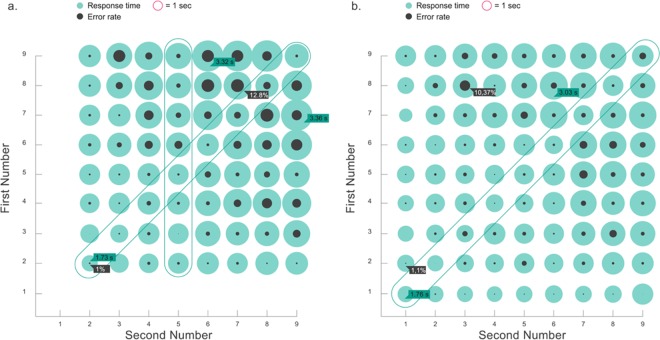
Average response times (green) and error rates (grey) for 1-digit multiplications and additions (Fig 7A and 7B, respectively). The diameters of each circle represent the magnitude of the measure. Larger numbers are more difficult, resulting in longer RTs and larger error rates, with the exception of the equal-numbers multiplications and additions, and multiplications by 5, highlighted in the figure.

As predicted by standard models, all the main effects that result in an increase of RT also results in an increase in the errors. Just for visualization purposes, we present in Fig 7 these effects in a compact manner for the times and addition tables. We performed a linear mixed effects analysis of the relationship between error rates and size and show in Table 3 the results of the mixed model linear regression. As an example, the expected error rate for the operation 7+8 is 1.6 + 0.071x56 (56 is the product of the operands), i.e., 5.58%.

**Table 3:** results of the linear regressions for error rates (in %) using the product of the operands as independent variable.

ADDITION	intercept [%]	std error	t-value	slope [%/u]	Slope std error	t-value
**Different Operands**	1.6	0.3	5.6	0.071	0.007	9.5
**Tie Problems**	1.2	0.6	2.1	0.051	0.013	3.9
**MULTIPLICATIONS**	**intercept [%]**	**std error**	**t-value**	**slope [%/u]**	**Slope std error**	**t-value**
**Different Operands**	1.56	0.46	3.4	0.211	0.008	24.6
**Tie Problems**	2.83	0.65	4.3	0.06	0.01	4.8

**Fig 8:**
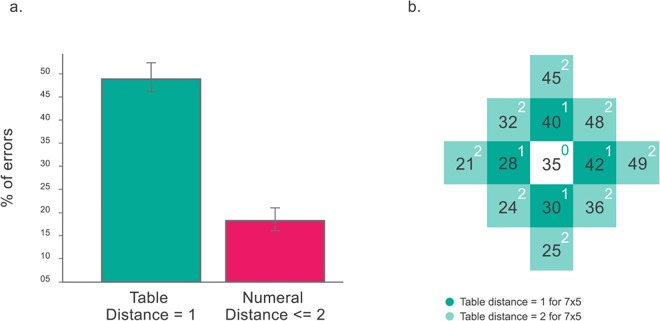
(a) Most errors correspond to one of the four possibilities given by a table distance = 1. For a fair comparison against a possibility with four different results, we also plot the errors corresponding to numerical distance < = 2. Inter-subjects SEs are also shown. (b) Figure explaining the concept of table distance for the 7x5 problem. The numbers in dark green are separated by a table distance of 1 and in light green by 2.

Also in consistency with current models and with lab based observations (see a review in [8]) we observed that the most commons errors (43%) correspond to a neighbor in the times table (this concept is visually explained in Fig 8B). A direct comparison between neighbors of the multipliers in the times table vs numerical neighbors to the response in the number line showed that the former were much more frequent, a highly significant result as revealed by a chi-squared test (p<5.29e-010) (Fig 8A).

### 6. New Results on Mathematical Cognition: Arithmetic, Learning and Phonology. Order Effect by Problem

Our results confirm theoretical predictions [8] that there is an order effect both in additions and multiplications. In our study the order effect shows that on average the problem is easier when the first operand is larger (i.e. 7 x 4 is faster than 4 x 7). In a study in China, where tables are learned almost as a poem, in a specific order in which the smallest operand is mentioned first (four times seven is twenty-eight) it was shown that problems in this order were responded more rapidly and with less errors. This suggests, more generally that there may be an effect of phrasing of mathematical problems that may underlie arithmetic abilities. In particular, one standard way in which a problem may be biased towards an order in its phrasal presentation is when it rhymes. For instance, *“Six times eight*, *equals forty-eight*” rhymes, but “*Eight times six*, *equals forty-eight”* does not.

Of all one digit multiplications, only four can be pronounced in a rhymed verse (in Spanish) when pronounced in the proper order. Those whose last digit of the result equals the second multiplier and when the result is larger than 20: 7 x 5 = 35, 9 x 5 = 45, 6 x 4 = 24 and 6 x 8 = 48 (note that 3 x 5 = 15 and 6 x 2 = 12 do not rhyme). Hence, a working hypothesis is that these specific problems will 1) show the largest order effect and 2) the effect will be such that the problem in the rhyme order will be performed faster.

**Fig 9:**
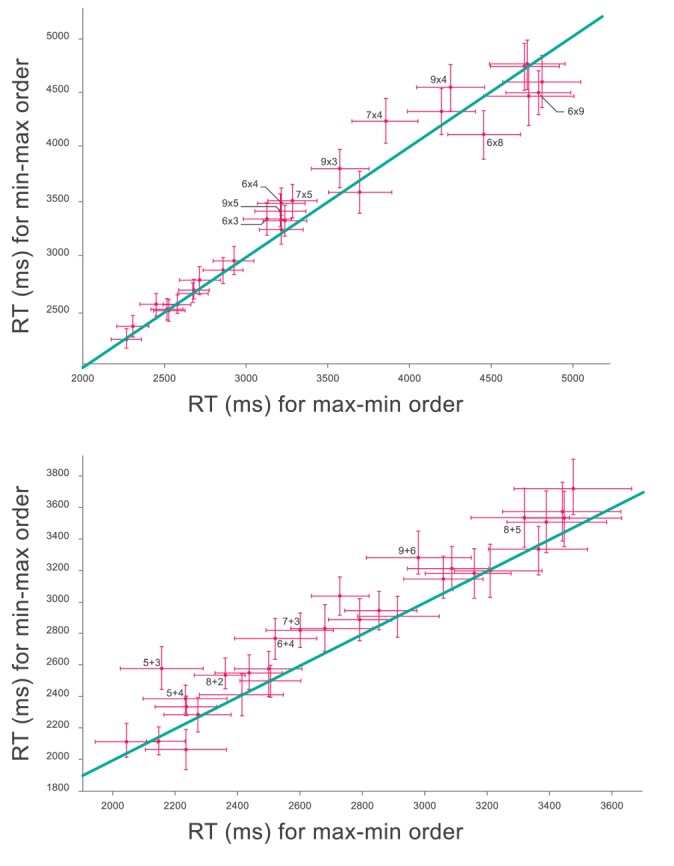
mean RTs and standard error for Op1>Op2 vs. the same for Op1<Op2, for the times table (above) and addition table (below). All but tie cases of the tables are shown, but just those in which the difference between both RTs at the two axes is significant are labeled; these are the points which standard error bars do not cross the diagonal line.

In our study we were in a good position to examine this hypothesis, because we had substantial data that could allow us to inquire the order effect for each individual problem (Fig 9).

In the times table, by the likelihood ratio tests, including or not the fixed effect Op1>Op2 in the mixed linear model, we found 5 problems which showed a significant (p<0.05) order effect (notation indicates the order in which the problem is performed faster):

[9×4,6×8,7×4,7×5,6×4]

Note that three of the four problems for which there is a rhyme show a significant order effect and in all of them, as predicted by the phonological hypothesis, the problems in the rhyme order is performed faster. We also note the main effect described above: 4 out of these 5 problems follow the rule that problems are faster when the first multiplier is larger.

Repeating the same analysis with a lower probability threshold shows that this pattern is very reliable. There are 9 problems showing an order effect with the very lax p value of 0.1. In the resulting list [**9 x 5**, 9 x 4, 9 x 3, **7 x 5**, 7 x 4, **6 x 4**, 6 x 3, **6 x 8**, and 8 x 9] the four problems for which there is a rhyme show a significant order effect and in all of them, as predicted by the phonological hypothesis, the problem in the rhyme order is performed faster. The main effect of Op1 > Op2 is found in 7 of these 9 problems.

An alternative interpretation to a rhyme effect is that the effect is instead due to the fact that the last digit of the result equals the second multiplier. We used as controls the problems 3 x 5 = 15 and 6 x 2 = 12, which don’t rhyme but their answer’s last digit equals their second multipliers. Interestingly, the problems 3 x 5 and 6 x 2 were answered in average faster than 5 x 3 and 2 x 6, as predicted, but none of these two problems showed a significant order effect (p = 0.22 and p = 0.50, respectively). As the data stands it is not possible to discard the hypothesis that some kind of priming mechanism is at play instead of a rhyming effect.

For additions, the phonological hypothesis cannot be examined (because one digit additions are never phrased in rhymes). We nevertheless performed the same analysis and identified 2 problems with significant order effect. Both satisfy the max-min order (9+6, 5+3). Relaxing the threshold to 0.1 resulted in 6 problems with significant order effect (9+6, 8+5, 8+2, 7+3, 6+4, 5+4, 5+3), all of them satisfying the Op1 > Op2 rule.

## 7. Discussion and Conclusion

Here we showed the validity of a research program in arithmetic based on large scale data sampling through mobile devices. Our results confirm, on a large sample, six of the main principles derived in a long tradition of investigation: size effect, tie effect, size-tie interaction effect, five-effect, RTs and error rates correlation effect, and most common error effects.

Our large dataset allowed us to perform an analysis of order effects for each individual problem. This was an aspect of the model for which there were controversial findings. Current theoretical models [8] predict an order effect, but without clear specification of what this order should be. For addition problems, one study in Canada found that the order effect was dominated by a max-then-min structure (i.e. 7 + 4 is easier than 4 + 7). This result is predicted by models in which additions are performed as a translation [16] starting from the first addend, with a distance given by the second addend. The smaller the second addend, the shortest the translation. An alternative interpretation, however, is that only half of arithmetic facts is stored in memory, and that the other half is retrieved by reordering the operands [10]. Indeed, results in China, where multiplications tables are phrased in one specific order, showed that multiplications (presented symbolically) are easier when they respect the phrasal structure in which they were verbally memorized [9]. This is in-line with the triple-code model of arithmetic [17] which proposes that arithmetic facts are stored as rote verbal phonological phrases, in a specific word order.

Here we had an ideal dataset to examine a prediction of this model, namely, that problems that can be pronounced in a rhymed verse will 1) show the largest order effect and 2) the direction of the effect will be such that problems presented in the rhyme order will be performed faster. Results confirmed these two predictions for the four problems that are phrased in a rhyme.

We also observed an order effect for multiplication that showed a max-then-min pattern. One possible explanation for this is that calculation is easier when performing fewer additions of the largest number (i.e. 8 x 3 is easier to compute as 8 + 8 +8 than as 3 + 3 +… + 3). In Spanish the linguistic expression for 8 x 3 is "8 por 3" which translates to 8 + 8 + 8. This expression, which is similar to "8 multiplied by 3" is the opposite expression than in English where "8 times 3" means literally 3 + 3 +… + 3.

To confirm that 1) "8 por 3" is interpreted as 8 + 8 + 8 and 2) that it is easier when performing fewer additions of the largest number, we conducted two queries through massive data gathering in twitter using our account (@ElGatoyLaCaja). In one query, we asked participants for the literal interpretation of A x B and in the other we asked how they organize this operation mentally.

In the first query we asked “What do you think ‘6 por 3’ means? We are not asking how do you solve it, but what do you think it literally means”. For the answer, two choices were given: “6 + 6 + 6” and “3 + 3 + 3 + 3 + 3 + 3“. A total of 915 people participated: 80% answered “6 + 6 + 6” and 20% the other option (p < 2e-78, according to a two-tailed binomial test, the null hypothesis being that both answers have equal probability). We repeated the same poll after some hours but asking for the literal interpretation of “3 por 6” instead of “6 por 3”. The results were consistent with the previous poll: 1080 people participate, 55% answered “3 + 3 + 3 + 3 + 3 + 3” and 45% the other option (p = 0.001, according to a two-tailed binomial test). This confirms that “A por B” is mostly interpreted as A repeated B times.

Then we studied how participants organize these problems mentally, independently of the literal interpretation of the question. In one poll we asked: “When you compute 7 x 4, what do you imagine:”. Three answers were enabled: “7 + 7 + 7 + 7”, “4 + 4 + 4 + 4 + 4 + 4 + 4” and “It is indistinct”. A total of 1008 subjects participate, 73% answered the first option, 3% the second, and 24% the third (p < 9e-175, according to a two-tailed binomial test, neglecting the answers “it is indistinct”). We repeated the same poll hours later but replacing “7 x 4” by “4 x 7” in the question. A total of 1045 subjects participate, 68% answered the first option (i.e. “7 + 7 + 7 + 7”), 10% the second, and 27% the third (p < 3e-76, according to a two-tailed binomial test).

Results were consistent with our hypothesis: in Spanish A x B is interpreted as A summed B times and, independently of the presented order, most people preferentially organize this operation as the max operand repeated a number of times given by the min operand. This suggests that, for the Spanish speakers, when a multiplication is presented in the max-min order it is presented in the more natural and easier mental representation of the product used during the learning process. Future comparative studies should explore the hypothesis that this effect may be reversed in phrasal structures (like in English and French) in which 8 times 3 is interpreted as 3 + 3 +… + 3 (8 times).

Above and beyond these results, our study bares an important practical conclusion, as proof of concept, that participants can be motivated to perform substantial arithmetic training simply by presenting it in a gamified format. There are many similar initiatives that have not been so successful in achieving such an engagement and we can then ask, with the hopes of improving future similar initiatives, what made this experience successful. The space of games is very high dimensional and complex and hence there is no way for an individual study to come up with definite conclusions. However, we mention some aspects that in our experience turned out to be important: 1) an engaging gameflow, with difficulty levels carefully designed according to gaming best practices (as explained in Section 1); 2) the long-term goal of squaring large numbers, a classical operation for expert mental calculators that, because of its apparent difficulty, produces an hedonic feeling when achieved, and 3) the ‘collaborative science’ slogan used to launch the app, describing it as a new form of common-based peer production of scientific data (from the feedback obtained, many of the participants were much more engaged to participate knowing that it was clear that this project was a way to contribute to the construction of scientific knowledge), and the frequent posts regarding performance that were made, generating constant exchange between game programmers/designers and users as well as in between users.
